# Intervention to promote mental health and psychosocial support to promote social cohesion in the context of ongoing crisis and post conflict

**DOI:** 10.1192/j.eurpsy.2023.247

**Published:** 2023-07-19

**Authors:** E. Dozio

**Affiliations:** Action contre la Faim, Paris, France

## Abstract

**Introduction:**

Armed conflicts, collective situations of adversity, and gross social injustices cause widespread mental suffering in affected populations. In these crises, conflicts break down and traditional community support mechanisms are weakened or destroyed. The loss of trust in others and the lack of hope for change undermine social cohesion at the deepest levels of communities. Therefore, it is important not to overlook the psychosocial impacts of social injustice and violence on the individual and society undermines other efforts to build peaceful societies. Nevertheless, the use of mental health and psychosocial support (MHPSS) approaches to support social cohesion is still very uncommon.

**Objectives:**

The objective of the proposed intervention in the Ituri province of the Democratic Republic of Congo was to complement the economic recovery activities of the most vulnerable populations with a psychological support approach. This was to ensure more sustainable results in the appropriation of problem management strategies through the strengthening of individual well-being and group support mechanisms.

**Methods:**

The psychosocial intervention is organized around a community psycho-education to sensitize the populations to mental health issues and to promote the awareness of their possible suffering in order to access a psychological care system. The protocol included five weekly group sessions designed to strengthen participants’ individual and collective psychological resources. Several indicators were measured to assess the impact on social cohesion (psychological well-being, psychological resilience, prosocial behavior, etc.)

**Results:**

In eight months of intervention between July 2021 and February 2022, 1024 people were able to participate in the psychological support program. 90% of them showed improvement in psychological well-being, daily functioning and resilience. In addition to these very optimistic results on individual aspects, 65% of the participants increased the level of prosocial behaviour.

**Conclusions:**

The psychosocial intervention proposed in an area of permanent conflict and adversity was mainly aimed at improving the well-being of people showing signs of distress to make them better able to complete their economic activity project. The results showed that taking into account the psychosocial dimension, not only reduced distress and allowed people to better project themselves in the future, but also promoted prosocial behavior. All these elements contribute strongly to social cohesion.

**Disclosure of Interest:**

None Declared

